# LINC01119 negatively regulates osteogenic differentiation of mesenchymal stem cells via the Wnt pathway by targeting FZD4

**DOI:** 10.1186/s13287-022-02726-1

**Published:** 2022-01-29

**Authors:** Hongwei Gao, Hui Dong, Jiachun Zheng, Xufeng Jiang, Mingzhi Gong, Le Hu, Jinshan He, Yongxiang Wang

**Affiliations:** 1 Department of Orthopedics, Clinical Medical College of Yangzhou University, Northern Jiangsu People’s Hospital, No.98 of Nantong West Road, Guangling District, Yangzhou, 225001 People’s Republic of China; 2 Department of Graduate School, Dalian Medical University, No.9 of West Section of Lushun South Road, Dalian, 116044 People’s Republic of China; 3 Department of Trauma and Orthopedics, The Second Hospital, Cheeloo College of Medicine, Shandong University, No.247 of North Park Street, Jinan, 250033 People’s Republic of China

**Keywords:** LINC01119, Mesenchymal stem cell, Osteogenic differentiation, Wnt pathway, FZD4

## Abstract

**Background:**

Mesenchymal stem cells (MSCs) can differentiate into diverse cell types under specific conditions. Dysfunction in the osteogenic differentiation of MSCs can result in bone metabolism-related diseases, including osteoporosis. Accumulating evidence has revealed that long non-coding RNA (lncRNAs) play critical regulatory roles during MSC differentiation.

**Methods:**

In the present study, we identified an evolutionarily conserved lncRNA expressed during the osteogenic differentiation of MSCs, which we termed LINC01119. We first identified LINC01119 as a negative regulator of the osteogenic differentiation of MSCs.

**Results:**

LINC01119 knockdown markedly induced calcium deposition in bone marrow MSCs and promoted the osteogenic differentiation of MSCs. More importantly, we demonstrated the underlying molecular basis through which LINC01119 regulates osteogenesis via the Wnt pathway by targeting FZD4. Furthermore, we observed that transcription factor EBF3 could directly bind the promoter site of LINC01119.

**Conclusions:**

We first explored the molecular regulatory mechanism of LINC01119 during the osteogenic differentiation of MSCs and revealed that LINC01119 negatively regulates osteogenesis through the Wnt pathway by targeting FZD4.

**Supplementary Information:**

The online version contains supplementary material available at 10.1186/s13287-022-02726-1.

## Background

The human skeleton undergoes uninterrupted remodeling to maintain bone homeostasis, predominantly relying on a coordinated balance between bone resorption by osteoclasts and bone formation by osteoblasts [1, 2]. Mesenchymal stem cells (MSCs) can differentiate into diverse cell types under specific conditions, including chondrocytes, osteoblasts, and adipocytes [3, 4]. As one product of MSC differentiation, osteoblasts play a key role in bone formation and bone homeostasis [5]. Dysfunctional osteogenic differentiation of MSCs can lead to bone metabolism-related diseases such as osteoporosis [6, 7]. Therefore, further clarifying the molecular mechanism underlying MSC osteogenesis is crucial for comprehensively understanding bone diseases and facilitating clinical practice.

Reportedly, canonical Wnt signaling is critical for maintaining homeostasis and bone development in humans [8]. Transduction of Wnt signals through plasma membranes relies on the frizzled protein, a member of the G protein-coupled receptor family [9]. A study has indicated that FZD4 specifically activates the Wnt signaling pathway and promotes the osteogenic differentiation of MSCs [10]. Additional studies have revealed that the canonical Wnt/β-catenin pathway plays a vital role in the osteogenic differentiation of MSCs and bone metabolism [11].

Long non-coding ribonucleic acids (lncRNAs) are non-coding RNAs exceeding 200 nucleotides in length. Accumulating evidence has revealed that lncRNAs mediate cis or trans gene expression, thereby playing crucial roles in regulating numerous biological processes, such as embryonic development, bone formation, and cell differentiation [12]. Various lncRNAs have been extensively investigated and reportedly possess critical regulatory roles during MSC differentiation. LncRNA DANCR regulates gene expression by modulating epigenetic modification and can inhibit RUNX2 expression, thus suppressing the osteogenic differentiation of bone marrow MSCs (BMSCs) [13].

Herein, we investigated the roles of lncRNAs in regulating the osteogenesis of MSCs. We identified an evolutionarily conserved lncRNA expressed during MSC osteogenic differentiation, which we termed LINC01119. We first identified LINC01119 as a negative regulator of MSC osteogenic differentiation and observed that LINC01119 silencing markedly induced calcium deposition in MSCs and promoted the osteogenic differentiation of MSCs. Importantly, we demonstrated the molecular basis through which LINC01119 regulates osteogenesis via the Wnt pathway by targeting FZD4. Furthermore, we noted that transcription factor EBF3 could directly bind the LINC01119 promoter site.

## Methods

### Cell culture and phenotype identification

The human BMSCs (hBMSC) and the 293 T cell line were all purchased from BeNa Culture Collection (Beijing, China), maintained in DMEM (Dulbecco's modified Eagle’s medium) with 10% fetal bovine serum (FBS), 4 mM L-glutamine, and sodium pyruvate, and incubated in a 5% CO2 atmosphere at 37 °C. Then the cells were digested and incubated with specific antibodies for identification of surface markers. To verify the MSC, we selected human CD44-FITC and CD90 FITC (all from BD, San Jose, CA) as the antibodies for surface markers. A BD Influx cell sorter (BD) was used to perform flow cytometry.

### Gene expression profiles

We obtained the GSE80614 dataset from the National Center for Biotechnology Information Gene Expression Omnibus (GEO; https://www.ncbi.nlm.nih.gov/geo/). The matrix contained 66 samples, including osteogenic and adipogenic differentiation groups at different time points (0 h, 0.5 h, 1 h, 2 h, 3 h, 6 h, 12 h, 1 d, 2 d, 3 d, and 4 d), with each time point consisting of 3 cases. In the present study, we selected 0 h of osteogenic differentiation as the normal group and 12 h, 1 d, 2 d, and 3 d of osteogenic differentiation as osteogenic groups 1, 2, 3 and 4, respectively. R software (Version 3.3.6; The R Foundation for Statistical Computing, Vienna, Austria) and Bioconductor packages (impute and limma) were applied to analyze differentially expressed genes (DEGs) between osteogenic differentiation samples and normal samples. We identified the differentially expressed lncRNAs from them. Expression profiles were analyzed considering a P-value < 0.05 and log2 (fold change [FC]) > 2 as thresholds.

### DAVID analysis and gene set enrichment analysis (GSEA)

Next, we evaluated the underlying functions and some vital pathways involved in osteogenic differentiation. Accordingly, DAVID Bioinformatics Tool (https://david.ncifcrf.gov/) was employed to perform Gene Ontology (GO) and Kyoto Encyclopedia of Genes and Genomes (KEGG) analysis. Three categories were exported from the GO analysis results, namely, biological process, cellular component, and molecular function, with the *P*-value denoting the significance of the GO term enrichment among differentially expressed genes (*P* < 0.05). Pathway analysis was used to map genes to KEGG pathways, with the *P*-value denoting the significance of pathway correlations (*P* < 0.05).

### Prediction of target mRNA and transcription factor

On selecting the differentially expressed lncRNA, the potential target mRNA was analyzed by Cis and Trans Target Prediction, with the target mRNA then selected according to the KEGG results and the PPI network made by cytoscape. The potential transcription factor could be predicted in UCSC and the Lisa database after determining the lncRNA promoter region in NCBI; binding sites and motif diagrams of the transcription factor were predicted using the Jaspar database.

### Osteogenic differentiation assay

For osteogenic differentiation, hBMSCs were cultured in an osteoblast-specific induction medium, α-MEM supplemented with FBS, 10 mM glycerol-2-phosphate (Sigma-Aldrich, St. Louis, MO, USA), 20 mM L-ascorbic acid (Sigma-Aldrich), and 0.1 mM dexamethasone (Sigma-Aldrich). The culture medium was replaced every 3 d for 14 d. Then, cells were harvested and analyzed at 0 d, 7 d, and 14 d. Osteogenic differentiation was evaluated using quantitative real-time PCR (qRT-PCR), western blotting, alkaline phosphatase (ALP) activity assay, and alizarin red S (ARS) staining.

### Quantitative real-time PCR

Total RNA was extracted from BMSCs using TRIzol reagent, according to the manufacturers' instructions. Reverse transcription was performed using 1 μg of RNA. Real-time quantitative PCR was performed using a Master Mix kit (Promega Corporation). Relative changes in gene expression were assayed using the 2^−ΔΔCt^ method. Primers were designed and the sequences are as follows: LINC01119 (forward: CTGCTGGGCTGAAGGGACT; reverse: TGCCGAAGGAACCACGAC), RUNX2 (forward: CACAAGTGCGGTGCAAACTT; reverse: GACTCTGTTGGTCTCGGTGG), ALP (forward: GGGCATAGACTTCAACCA; reverse: CAGAGCCATCGTCCACCA), OSX (forward: TCCTGCGACTGCCCTAAT; reverse: GCGAAGCCTTGCCATACA), OCN (forward: AGGGCAGCGAGGTAGTGA; reverse: CCTGAAAGCCGATGTGGT), Wnt5α (forward:ATACCTTGAGCACGACGAA; reverse: TTGATGGCACTGTTTGGA), LRP5 (forward: GATGACCAGAGCGACGAG; reverse: GGAGGATGATGCCAATGAC),FZD4 (forward: TCCCACCACAGAACGACC; reverse: GCCAGCATCATAGCCACA).

### Western blot analysis

Cells were lysed on ice in Cell Lysis Buffer (Sigma-Aldrich) and denatured by heating at 95 °C for 15 min with the loading buffer (BioTNT). A 30-μg protein sample was used for SDS-PAGE electrophoresis. Proteins were transferred through electroblotting onto polyvinylidene fluoride membranes (Millipore). After blocking in non-fat milk for 60 min, membranes were incubated with primary antibodies at 4 °C for 12 h, followed by incubation with an HRP-labeled secondary antibody. The primary antibodies are as follows: RUNX2 (Abcam, ab76956), Wnt5α (Abcam, ab179824), LRP5 (Proteintech, 24899-1-AP), beta-catenin (Abcam, ab32572), β-actin (66009-1-Ig; Proteintech). Image Quant LAS 4000 (GE Healthcare) was used to confirm protein expression levels. β-actin expression was used as the control.

### Lentivirus-mediated overexpression and knockdown

The shRNA against human LINC01119 was cloned into a modified pLV-H1-Puro lentiviral vector. The corresponding sequences for sh LINC01119 were 5′‐GGATGAGCTCCAAGGTCTTAA‐3′(shRNA-1), 5′‐GCCATAGCTTGAGTAACATGT‐3′(shRNA-2), and 5′‐GGGCAATAAACCATGTGATTT‐3′(shRNA-3). The human LINC01119 was amplified using reverse transcription PCR and inserted into a modified pLV-EF1α lentiviral vector. FZD4 siRNAs (Thermo Scientific) were transfected using RNAiMAX transfection reagent (Thermo Scientific). The viral supernatant was used for infecting BMSCs. After 48 h, infected cells were selected using puromycin (2 mg/mL). Real-time PCR assays were performed to determine inhibition and overexpression efficiencies.

### Alizarin Red S staining and ALP activity detection

ARS staining was performed after 14 d to detect osteoblast calcification. Osteoblast cells were digested and seeded in 24-well plates, fixed in ethanol for 10 min, washed with distilled water three times, and then stained with the ARS staining solution (Cyagen, China), with the pH adjusted to 8.3 using HCl, for 30 min at 37 °C; photomicrographs were obtained to assess calcium nodule formation.

For ALP activity, cells were cultured in an osteoblast-specific induction medium for 7 d, rinsed with phosphate-buffered saline (PBS), fixed using paraformaldehyde, and incubated using the NBT/BCIP staining kit (CoWin Biotech, Beijing, China). ALP activity was assayed with the ALP Activity Kit (Biovision, Milpitas, CA) and normalized to the total protein content.

### Oil red O staining

Oil Red O staining was used to determine the extent of adipogenic differentiation of MSCs in this study. Briefly, isolated rat MSCs were seeded in 6-well plates at a density of 1.0 × 10^4^ cells per well in adipogenic medium. After washing three times with PBS, cells were fixed with ORO Fixative for 20 min and stained with 1% filtered Oil Red O (Amresco, Solon, OH) for 10 min. Then Mayer hematoxylin staining solution was used to restain the nucleus for 1–2 min. After washing with distilled water, the conversion of MSCs into adipocytes was confirmed by the detection of lipid droplets under microscopy (Olympus).

### MTT assay

The MTT assay was performed to detect osteoblast cell proliferation. Briefly, osteoblast cells (200 μL) were seeded in 96-well plates (6 × 10^3^/well). Cell viability was evaluated by adding 5 μg MTT (Sigma-Aldrich Corp., St. Louis, MO, USA) solution to each well of the cell culture plate, followed by incubation for 4 h. The medium was removed, and 150 μL of dimethyl sulfoxide was added to each well. Subsequently, the plate was agitated for 10 min on a shaker to dissolve formazan. Optical density was measured at 490 nm at different time points (0 d, 1 d, 2 d, and 3 d) using a microplate reader (Varioskan LUX, Thermo Fisher Scientific), followed by MTT curve construction.

### Electrophoretic mobility shift assay (EMSA)

The LightShift Chemiluminescent RNA EMSA Kit (Thermo Scientific) was used for electrophoretic mobility shift assay (EMSA) according to the manufacturers’ instructions. The biotin-labeled LINC01119 RNA probe was obtained by in vitro transcription using the Biotin RNA Labeling Mix (Roche). Unlabeled oligonucleotide was used as competitor probe.

### Chromatin immunoprecipitation (ChIP)

Chromatin immunoprecipitation (ChIP) assays were performed using the EZ-Magna ChIP A/G Assay kit (Millipore) according to the manufacturer’s instructions. Briefly, cells were crosslinked with 1% formaldehyde at 25 ± 5 °C room temperature for 10 min. Cells were then collected and lysed to isolate the nuclei with nuclear lysis buffer containing protease inhibitor cocktail (provided in the kit). Sonication was performed to shear chromatin, generating 200–1000 bp DNA fragments. The sheared chromatin was immunoprecipitated with primary antibodies, normal IgG, and anti-RNA polymerase II antibody (provided in the kit). After the protein-DNA crosslinking was reversed, the relative binding of EBF3 to the LINC01119 promoter was analyzed using PCR with an Applied Biosystems Simpliamp instrument (ThermoFisher, USA). Agarose gel electrophoresis was used to detect DNA–protein binding. Data were normalized to the input control.

### *Fluorescence *in situ* hybridization (FISH)*

Cells were cultured on the bottom of confocal dishes to achieve a 60–70% cell confluency. Next, cells were washed with PBS for 5 min and fixed with 4% paraformaldehyde at 25 ± 5 °C for 10 min. The cells were washed with PBS for 5 min, three times in total. Then, 1 mL precooled perfusate was added to each dish, and cells were maintained at 4 °C for 5 min. After discarding the perfusate, cells were washed with PBS three times, for 5 min each time. Each dish was premixed with 200 mL of pre-hybridized liquid (sealed at 37 °C for 30 min) and hybridized liquid (preheated at 37 °C); the prehybridization solution was discarded, and a suitable amount of probe hybridization solution containing the probe was added. The hybridization solution was sheltered from light overnight at 37 °C. Cells were washed three times using hybridized lotion I at 42 °C, for 5 min each time, and then once with hybridized lotion II and once with hybridized lotion III. Finally, cells were washed with PBS at 25 ± 5 °C. DAPI staining was performed for 10 min, followed by washing with PBS three times, for 5 min each time. Cells were observed using a confocal microscope.

### Statistical analysis

Data were analyzed using SPSS version 17.0 (SPSS Inc., Chicago, IL, USA) and GraphPad Prism 6 (GraphPad, La Jolla, CA). All data values are expressed as mean ± standard deviation (SD) of three independent experiments. Student’s t test or one-way analysis of variance (ANOVA) was used to determine differences between two groups. ANOVA followed by Tukey post hoc test was employed to compare more than three groups. P < 0.05 was considered statistically significant.

## Results

### Phenotype identification of hBMSCs

**Fig. 1:**
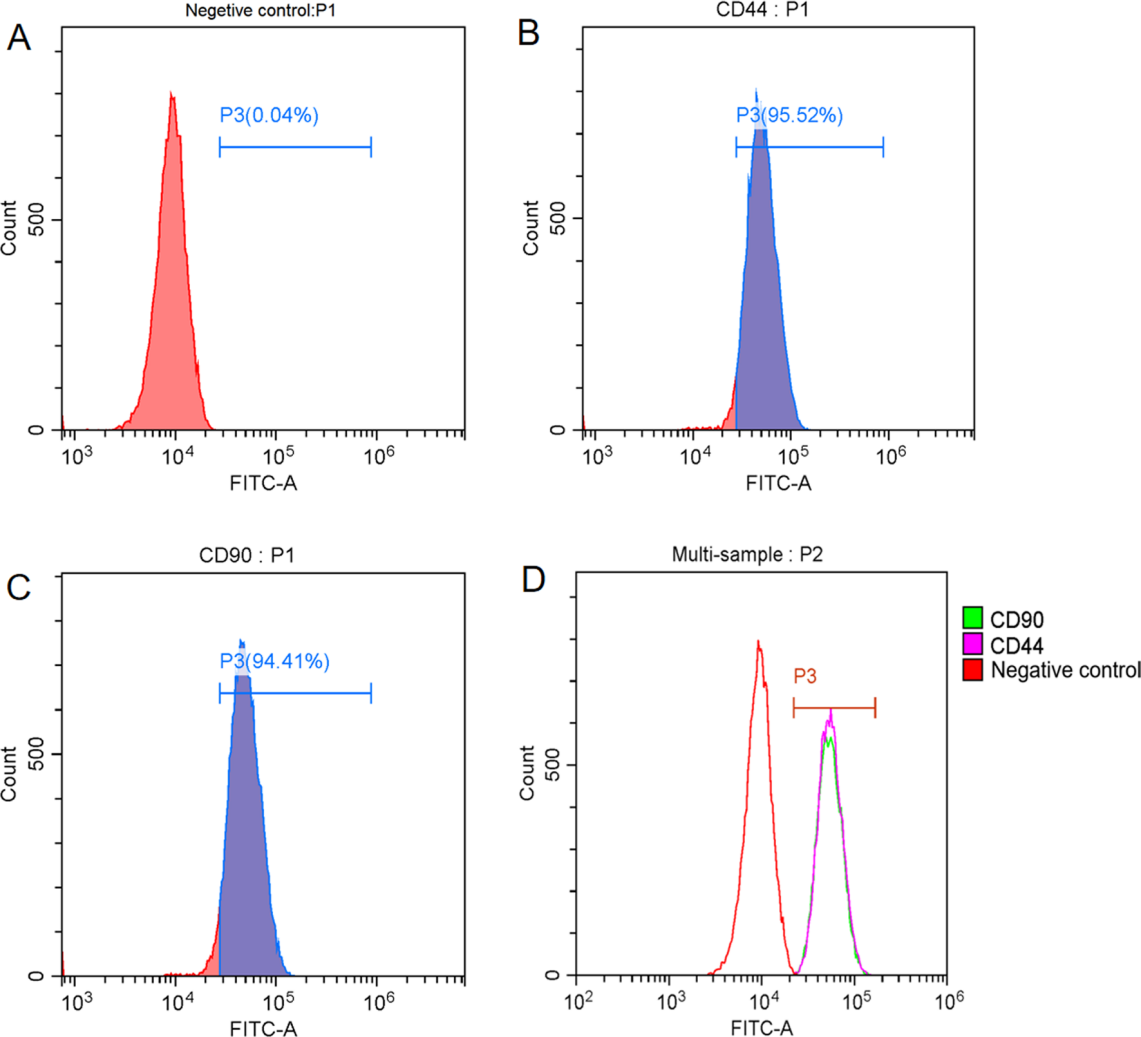
Phenotype identification of hBMSCs. the hBMSCs were positive for CD44 and CD90

To characterize purchased cells, we used flow cytometry to detect their phenotype and verify the MSC. We selected human CD44-FITC and CD90 FITC as the antibodies for surface markers. The results suggested that the hBMSCs were positive for CD44 and CD90 (Fig. 1).

### LINC01119 expression is downregulated during the osteogenic differentiation of BMSCs

**Fig. 2:**
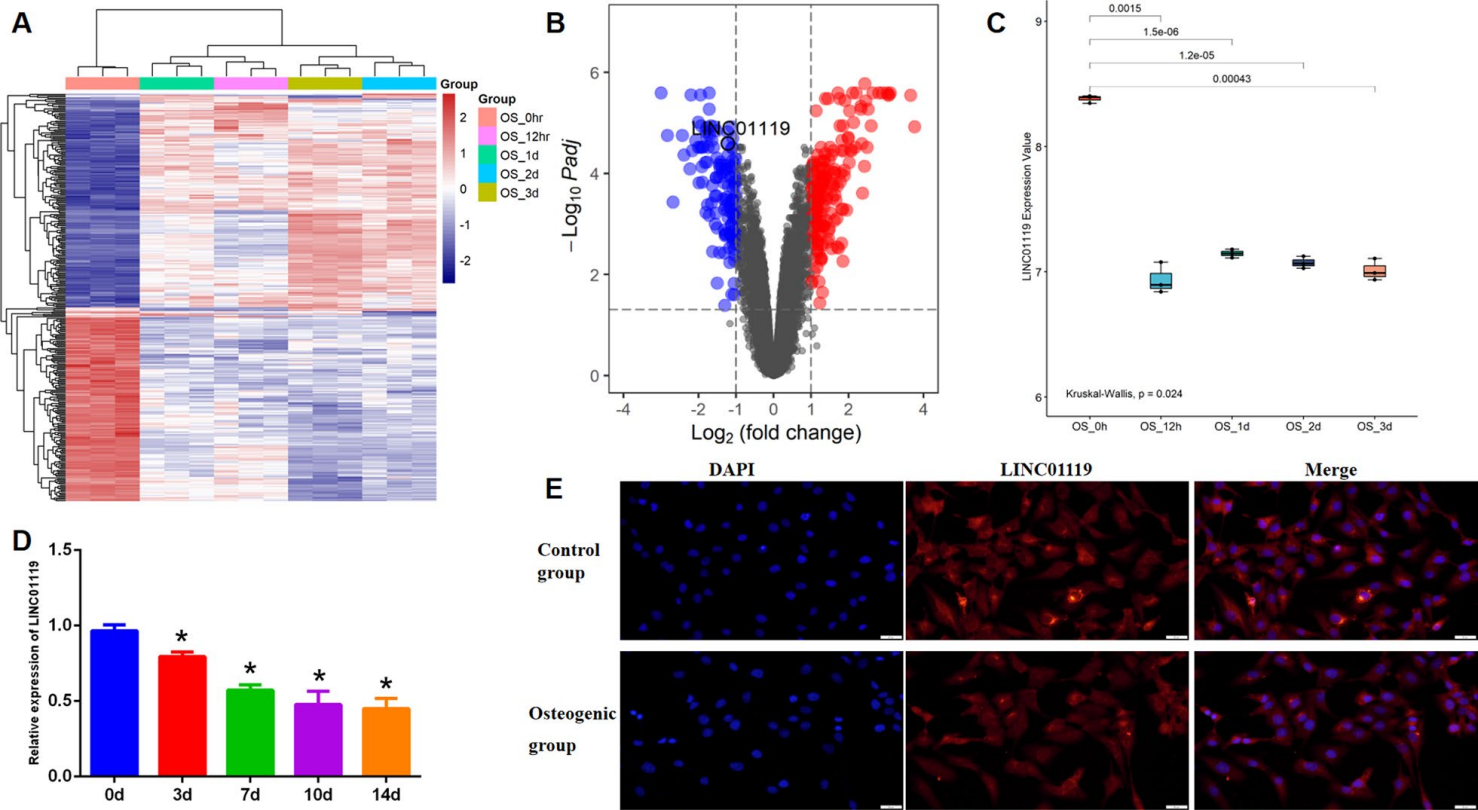
LINC01119 expression is downregulated during the osteogenic differentiation process of BMSCs. **A** Heat map of the top 100 DEGs at the differentiation time of 12 h, 1 d, 2 d or 3 d with that at 0 h. Red indicates higher expression, and blue indicates lower expression. **B** Volcano plot of DEGs at the differentiation time of 3 d with that at 0 h. Genes with significantly different expression are represented by red dots (upregulated) and blue dots (downregulated). Genes without significantly different expression are represented by gray dots. **C** Box plot of LINC01119 expression at differentiation time of 12 h, 1 d, 2 d or 3 d with that at 0 h. **D** qRT-PCR analysis for LINC01119 in osteogenic differentiation of BMSCs. **E** RNA-FISH results for LINC01119 in control group and osteogenic group

Herein, to study the gene expression profile at different time phases during the osteogenic differentiation of BMSCs, we analyzed the GSE80614 dataset. We compared differentially expressed genes (DEGs) at different differentiation time points (12 h,1 d, 2 d, and 3 d) with that at 0 h, identifying 592, 762, 1291, and 1793 DEGs in the four groups (Fig. 2A), and the lists of differentially expressed genes were submitted as supplemental files (see Additional file 1, Additional file 2, Additional file 3 and Additional file 4). We identified lncRNAs between the differentially expressed genes, and we found that LINC01119 was the common differentially expressed lncRNA and was downregulated (Fig. 2B, C). To further validate the reliability of the dataset, we assessed LINC01119 by qRT-PCR analysis to evaluate its role in the osteogenic differentiation of BMSCs. Interestingly, osteogenic induction significantly downregulated LINC01119 expression, suggesting the functional importance of LINC01119 in regulating osteogenesis (Fig. 2D). According to RNA-FISH results, LINC01119 was mainly located in the BMSC cytoplasm and was downregulated during osteogenic differentiation (Fig. 2E). Accordingly, we hypothesized that LINC01119 might be associated with the osteogenic differentiation of human BMSCs.

### LINC01119 function in the osteogenic differentiation of BMSCs

**Fig. 3:**
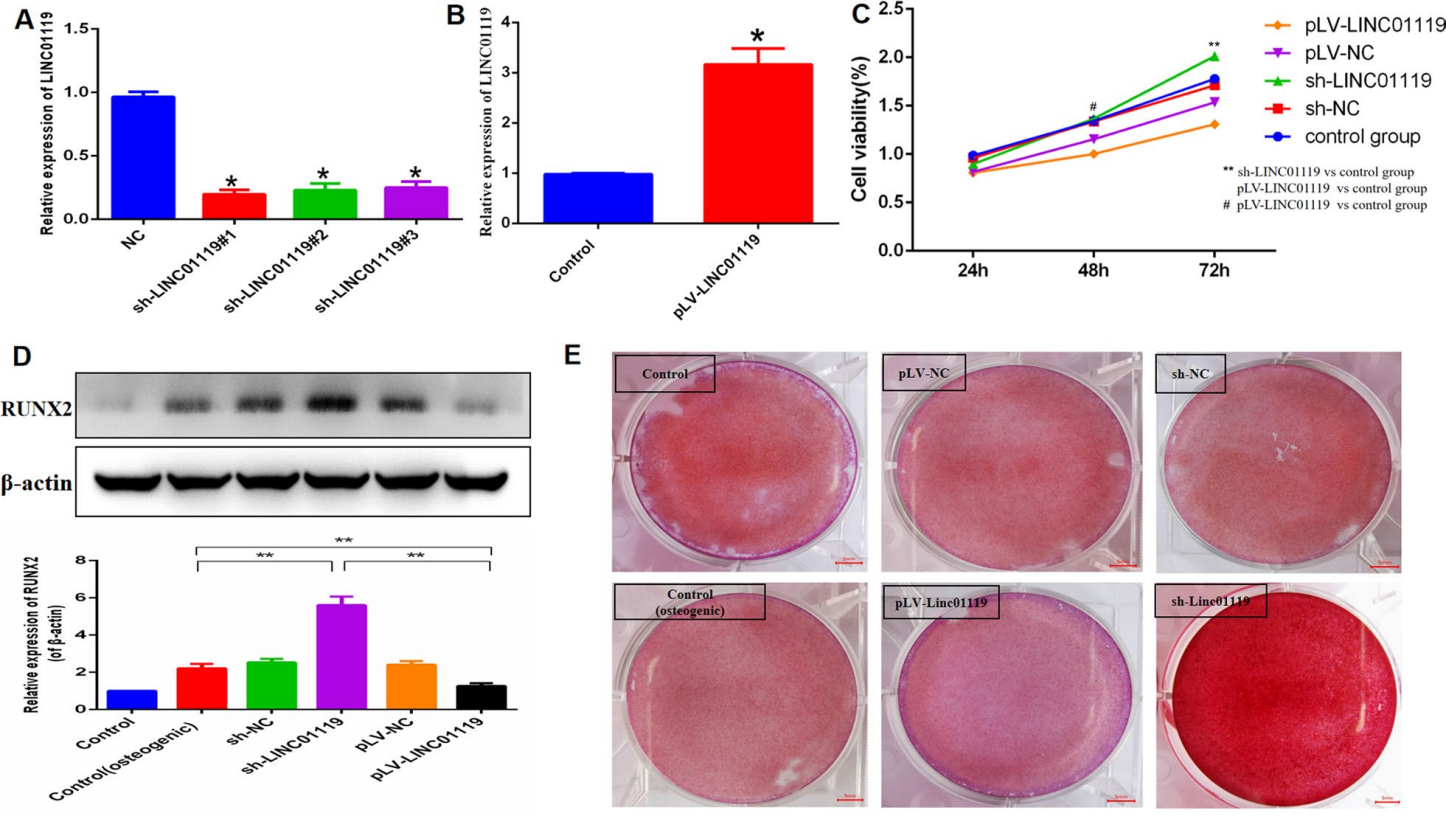
Knockdown of LINC01119 significantly promoted the osteogenic differentiation of MSC. **A**, **B** qRT-PCR analyses of the LINC01119 expression in BMSCs after transfection with sh-LINC01119 or pLV-LINC01119. **C** The proliferation ability of osteoblast cells after BMSCs transfected with sh-LINC01119 or pLV-LINC01119 on the 14th day by MTT assay. **D** Western blot analyses and quantitative analyses of the RUNX2 protein expression after transfection with sh-LINC01119 or pLV-LINC01119 on the 7th day. **E** Alizarin Red S staining for BMSCs transfected with sh-LINC01119 or pLV-LINC01119 after osteogenic induction media for 14 days

**Fig. 4:**
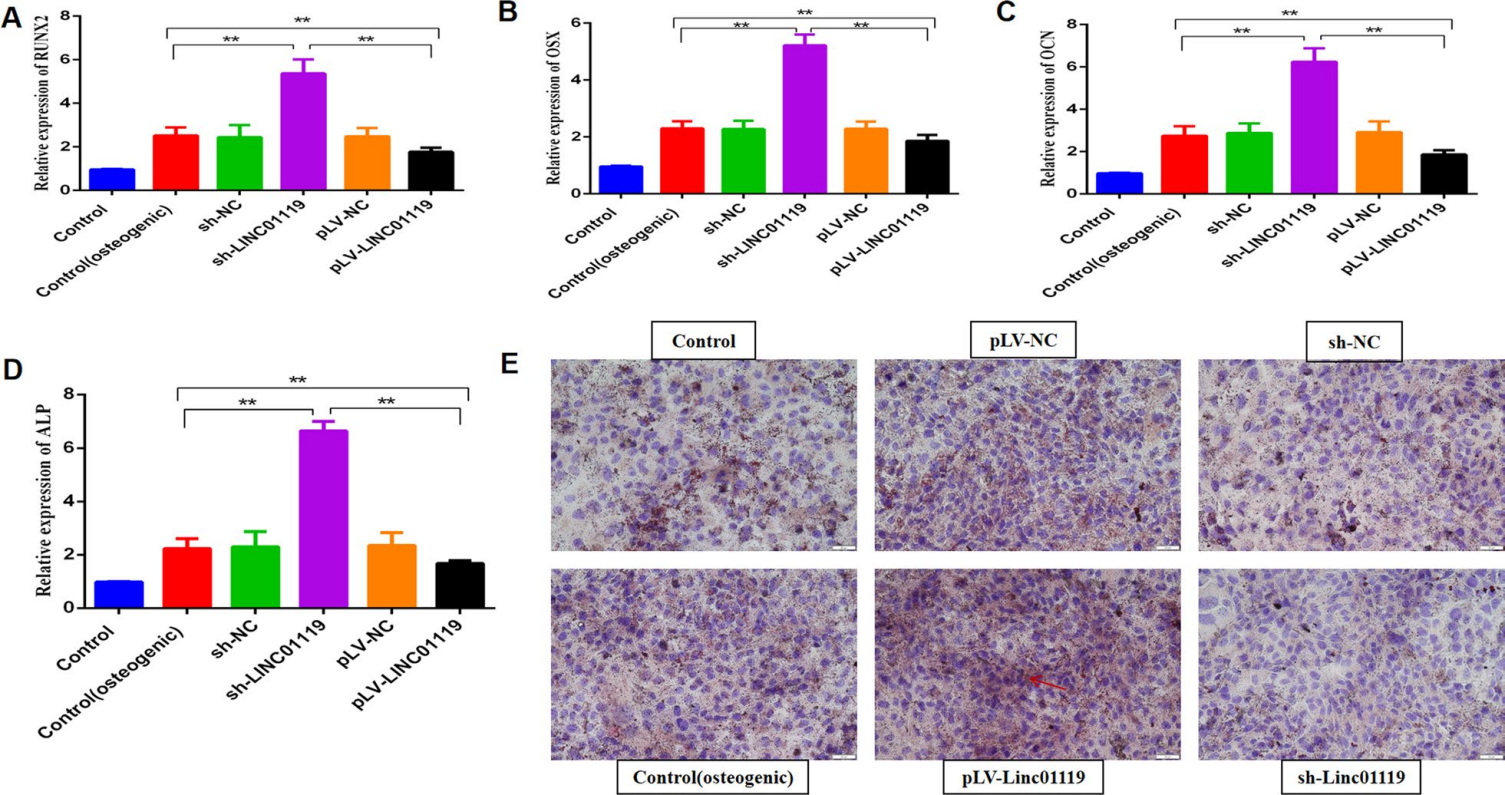
LINC01119 negatively regulated the osteogenic differentiation of MSC. **A**–**C** qRT-PCR analysis of RUNX2, OSX, OCN mRNA expression in BMSCs transfected with sh-LINC01119 or pLV-LINC01119 after 7 days of osteogenic media treatment. **D** ALP activity in BMSCs transfected with sh-LINC01119 or pLV-LINC01119 after 7 days of osteogenic media treatment. **E** Oil red-O staining for BMSCs transfected with sh-LINC01119 or pLV-LINC01119 after adipogenic induction media for 14 days; the arrow indicates the fat formation in brown areas

To examine whether LINC01119 plays a critical role in the osteogenic differentiation of BMSCs, we used a specific shRNA to silence LINC01119 and lentivirus infection to overexpress LINC01119 transcripts on 0 d. We designed three shRNAs targeting LINC01119 and selected the most efficient shRNA(sh#1) for the subsequent experiments to control potential off-target effects, using qRT-PCR to determine the efficacy (Fig. 3A, B). The effect of LINC01119 inhibition on osteoblast cell proliferation was evaluated using the MTT assay. The proliferation of osteoblast cells in the sh-LINC01119 group was significantly increased (Fig. 3C). Next, ARS staining was performed after treating BMSCs with osteogenic induction media for 14 d. LINC01119 depletion markedly induced calcium deposition in BMSCs (Fig. 3E). Several osteogenic markers were assessed at mRNA and protein levels after 7 d of osteogenic media treatment. LINC01119 knockdown significantly increased the protein expression of RUNX2 (Fig. 3D) and the mRNA expression levels of RUNX2, OSX, OCN (Fig. 4A, B, C), while enhancing ALP activity (Fig. 4D). In contrast, high LINC01119 expression significantly inhibited the mineralized nodule formation in BMSCs (Fig. 3E) and the RUNX2 protein expression (Fig. 3D). LINC01119 upregulation suppressed mRNA expression levels of RUNX2, OSX, and OCN and ALP activity (Fig. 4A, B, C, D). Furthermore, we observed that LINC01119 upregulation increased adipose tissue formation, whereas its downregulation decreased adipose tissue formation, as assessed using Oil red-O staining (Fig. 4E). Accordingly, we hypothesized that LINC01119 plays a critical role in human BMSC differentiation.

### *LINC01119 regulates BMSC osteogenic differentiation *via* Wnt signaling*

**Fig. 5:**
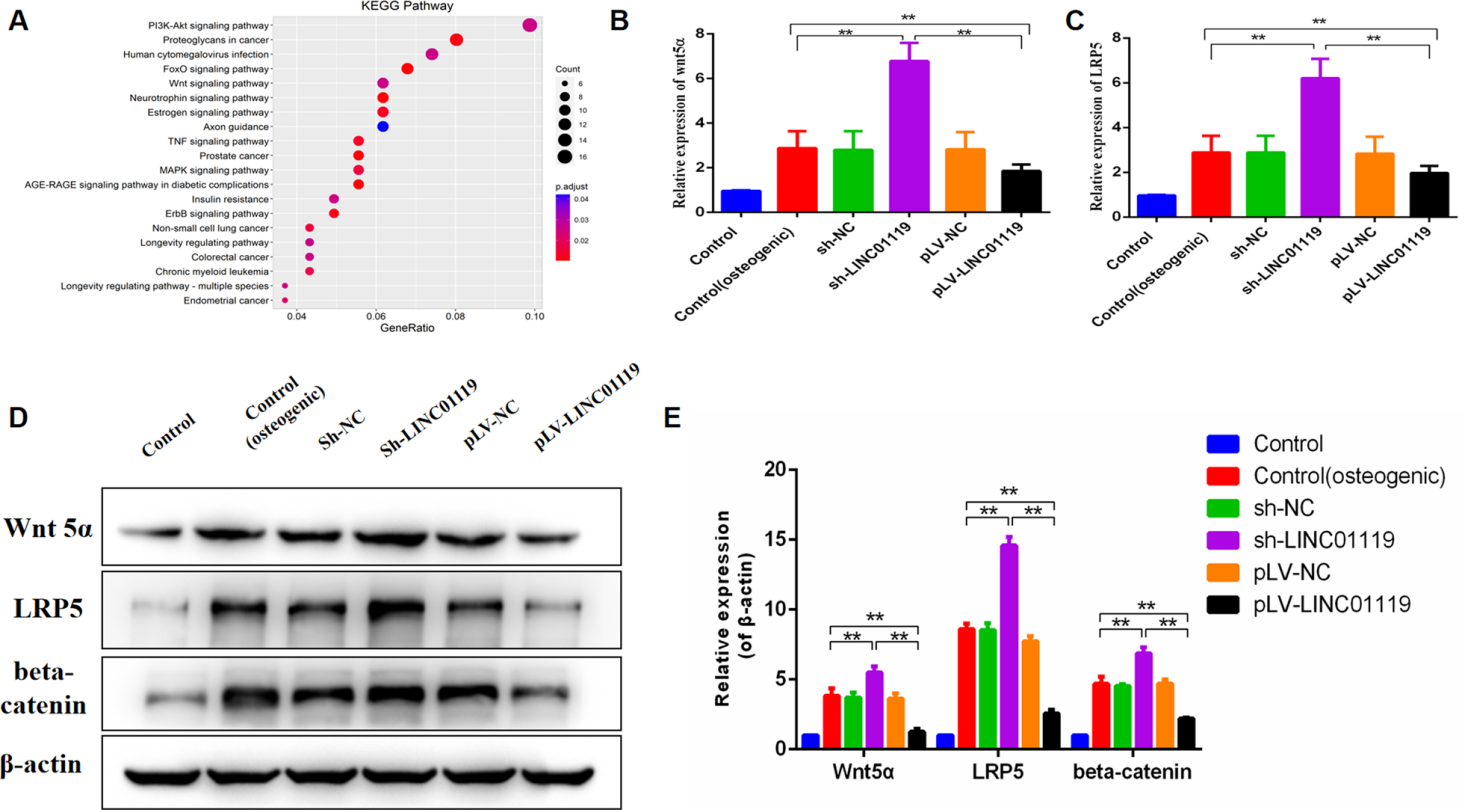
LINC01119 regulates the BMSCs osteogenic differentiation via the Wnt signaling. **A** Top 20 significant enrichment KEGG pathways in the dataset GSE80614 by DAVID. **B**, **C** qRT-PCR analyses of Wnt5α and LRP5 mRNA expression in BMSCs transfected with sh-LINC01119 or pLV-LINC01119. **D**, **E** Western blot analyses and quantitative analyses of the Wnt5α, LRP5 and beta-caten in protein expression after transfection with sh-LINC01119 or pLV-LINC01119

To explore the mechanism through which LINC01119 regulates BMSC osteogenic differentiation, we used DAVID for the KEGG pathway analysis of the GSE80614 dataset. We identified that Wnt, PI3K/AKT, and MAPK signaling pathways are involved in osteogenic differentiation (Fig. 5A) and selected the Wnt signaling pathway for further validation.

**Fig. 6:**
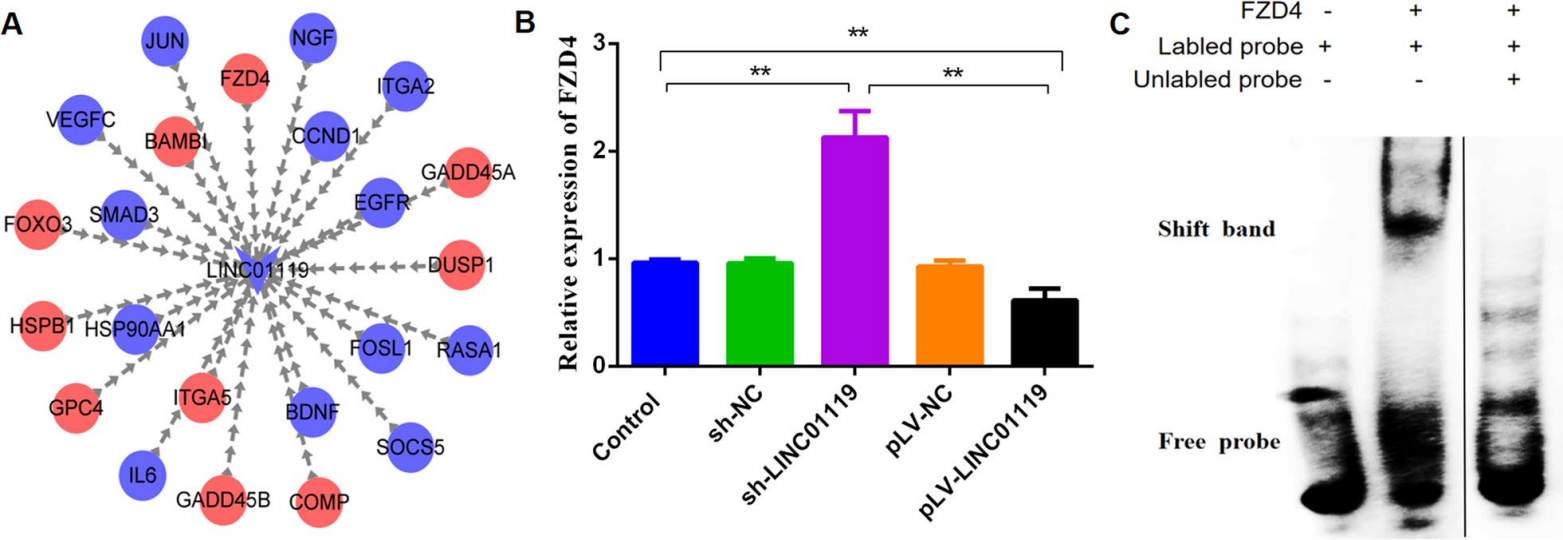
LINC01119 might interact with FZD4. **A** PPI network of the target genes of LINC01119 by Cis and Trans analyses. Red indicates upregulated expression, and blue indicates downregulated expression. **B** qRT-PCR analyses of FZD4 mRNA expression in BMSCs transfected with sh-LINC01119 or pLV-LINC01119. **C** RNA-EMSA assay of LINC01119-FZD4 interactions, the result demonstrated LINC01119 can interact with FZD4. The gel was cropped from different parts of the same gel, and the complete gel was submitted as Additional file 6

To determine whether LINC01119 influenced osteogenesis via Wnt signaling, we used the shRNA(sh#1) to silence LINC01119 and used lentivirus infection to overexpress LINC01119 transcripts on 0 d. We then assessed several markers in the Wnt pathway at mRNA and protein levels after 7 d of osteogenic media treatment. LINC01119 knockdown significantly increased mRNA and protein expression levels of Wnt5α, LRP5 and beta-catenin (Fig. 5B, C, D, E). And we also assessed several markers in the PI3K and MAPK pathway at protein levels, knockdown and overexpression of LINC01119 had slight effect on protein expression levels of p-mTOR and p-JNK (see Additional file 5). Hence, LINC01119 might regulate the osteogenic differentiation of BMSCs via Wnt signaling. Next, Cis and Trans analyses were performed to predict target genes of LINC01119. Overall, 10 mRNAs were upregulated, whereas 13 were downregulated (Fig. 6A). We selected FZD4 in the Wnt signaling pathway for further evaluation. Based on the RT-PCR findings, the mRNA expression of FZD4 increased following LINC01119 downregulation (Fig. 6B). And then we performed the EMSA using the LINC01119 probes. As shown in Fig. 6C, when RNA probes were incubated with FZD4, a specific RNA-protein complex was observed; these demonstrated that LINC01119 can interact with FZD4 (see Additional file 6, Additional file 8A).

### LINC01119 negatively regulates FZD4 in the osteogenic differentiation of BMSCs

**Fig. 7:**
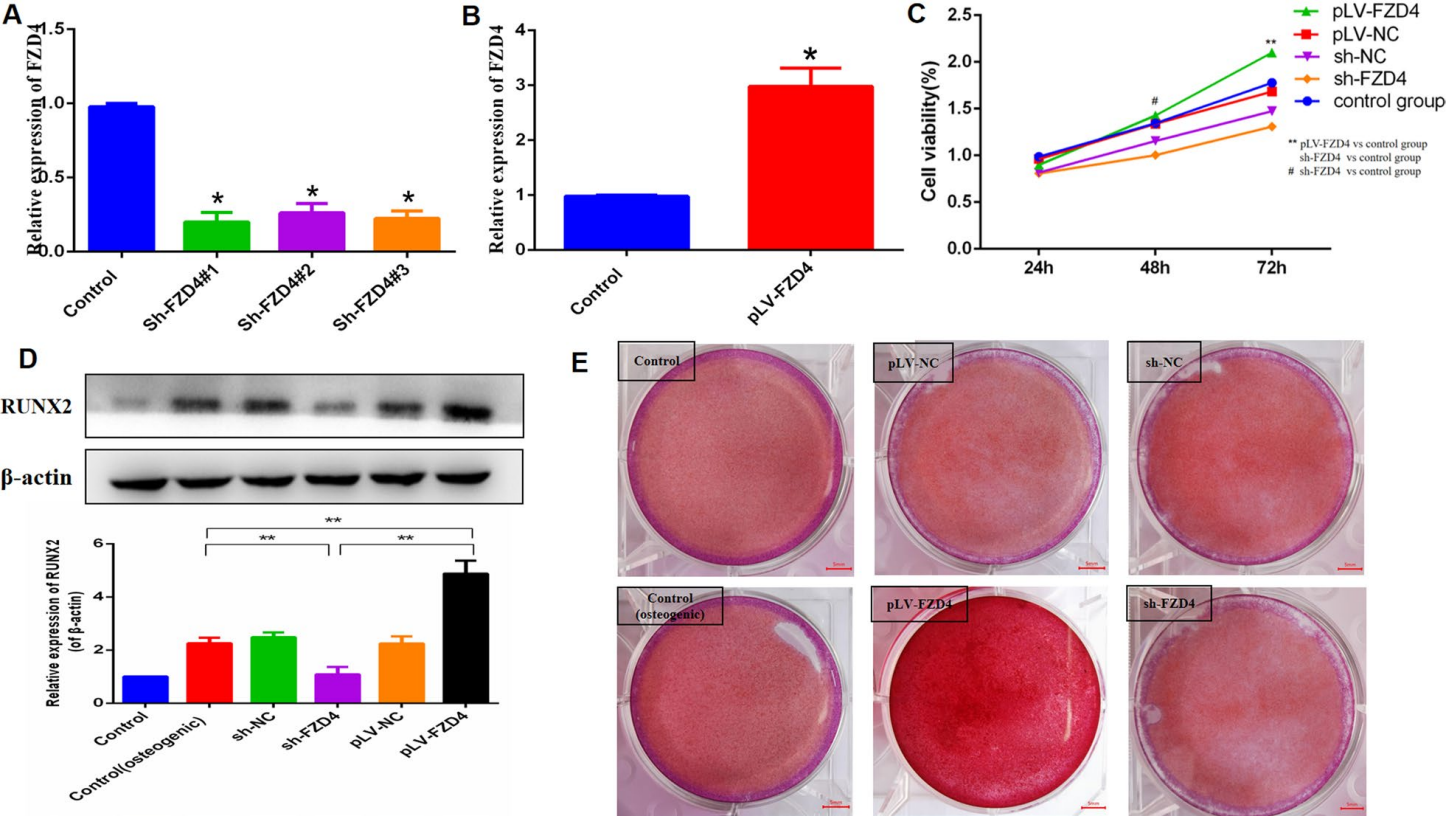
FZD4 significantly promoted the osteogenic differentiation of MSC. **A**, **B** qRT-PCR analyses of the FZD4 expression in BMSCs after transfection with sh-FZD4 or pLV-FZD4. **C** The proliferation ability of osteoblast cells after BMSCs transfected with sh-FZD49 or pLV-FZD4 on the 14th day by MTT assay. **D** Western blot analyses and quantitative analyses of the RUNX2 protein expression after transfection with sh-FZD4 or pLV-FZD4 on the 7th day. **E** Alizarin Red S staining for BMSCs transfected with sh-FZD4 or pLV-FZD4 after osteogenic induction media for 14 days

**Fig. 8:**
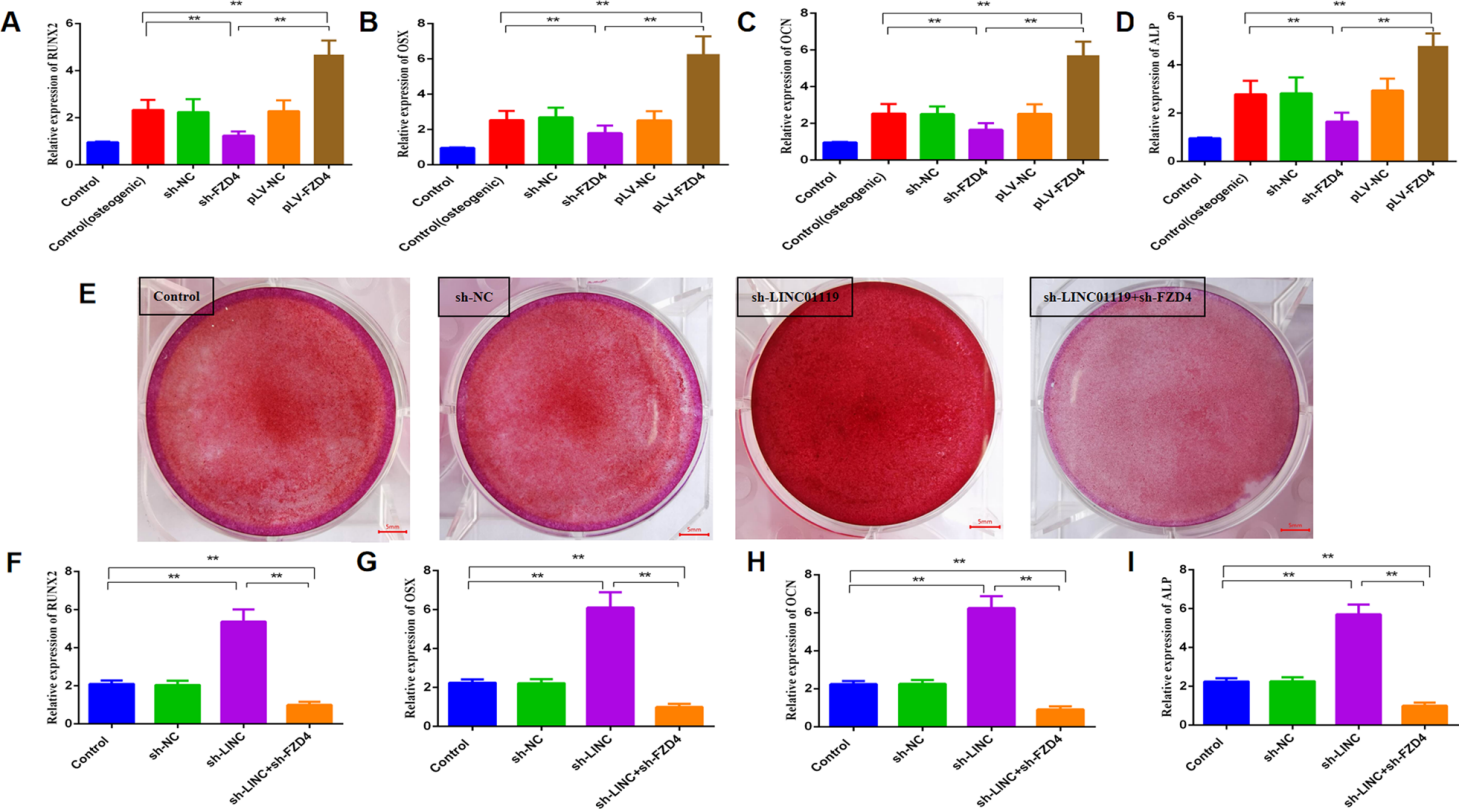
LINC01119 negatively regulates FZD4 in osteogenic differentiation of BMSCs. **A**–**C** qRT-PCR analyses of RUNX2, OSX, OCN mRNA expression in BMSCs transfected with sh-FZD4 or pLV-FZD4 after 7 days of osteogenic media treatment. **D** ALP activity in BMSCs transfected with sh-FZD4 or pLV-FZD4 after 7 days of osteogenic media treatment. **E** Alizarin Red S staining for BMSCs transfected with sh-LINC01119 and sh-FZD4 in the meantime after osteogenic induction media for 14 days. **F**–**H** qRT-PCR analyses of RUNX2, OSX, OCN mRNA expression in BMSCs transfected with sh-LINC01119 and sh-FZD4 after 7 days of osteogenic media treatment. **I** ALP activity in BMSCs transfected with sh-LINC01119 and sh-FZD4 after 7 days of osteogenic media treatment

To examine the role of FZD4 in the osteogenic differentiation of BMSCs, we used a specific shRNA to silence FZD4 and used lentivirus infection to overexpress FZD4 transcripts. We designed three shRNAs targeting FZD4 and selected the most efficient shRNA(sh#1) for subsequent experiments. qRT-PCR was used to determine the efficacy (Fig. 7A, B). High FZD4 expression markedly promoted osteoblast cell proliferation (Fig. 7C) and induced calcium deposition in BMSCs (Fig. 7E). FZD4 overexpression significantly increased RUNX2 protein expression (Fig. 7D), as well as the mRNA expression levels of RUNX2, OSX, and OCN (Fig. 8A, B, C). Moreover, FZD4 overexpression enhanced ALP activity (Fig. 8D).

To examine whether LINC01119 modulates the expression of FZD4, a crucial regulator in the Wnt signaling pathway, we utilized specific shRNAs to silence LINC01119 and FZD4 simultaneously. We observed that low LINC01119 expression markedly induced calcium deposition in BMSCs; however, FZD4 knockdown could alleviate the calcium deposition effect mediated by LINC01119 (Fig. 8E). Likewise, FZD4 knockdown suppressed the promoting effect on the mRNA expression levels of RUNX2, OSX, and OCN and ALP activity induced by LINC01119 depletion (Fig. 8F, G, H, I). Collectively, our findings revealed that LINC01119 negatively regulates FZD4 during the osteogenic differentiation of BMSCs.

### EBF3 acts as the transcription factor of LINC01119

**Fig. 9:**
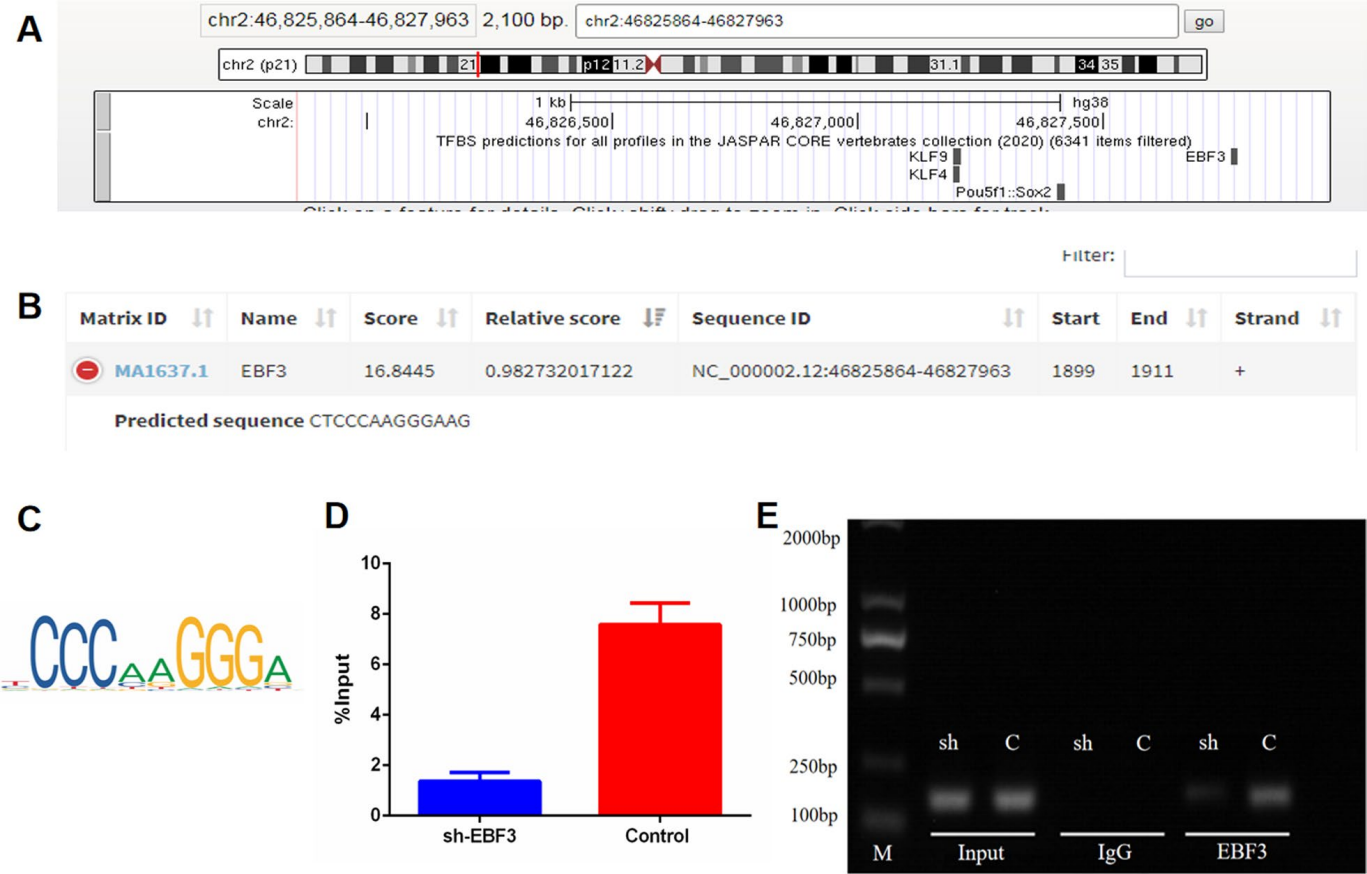
EBF3 was the transcription factor of LINC01119. **A** The potential transcription factors for LINC01119 were predicted in UCSC data, and the top four transcription factors were KLF9, KLF4, SOX2 and EBF3. **B** The potential transcription factors predicted by Lisa database was EBF3. **C** Motif diagrams of EBF3 predicted by Jaspar database. **D**, **E** Chromatin immunoprecipitation (ChIP) assay for EBF3 and LINC01119, the result demonstrated EBF3 directly regulates LINC01119 expression, knockdown of EBF3 supressed the binding of ENF3 and LINC01119, and the band became unconspicuous

To explore the expression mechanism underlying LINC01119, we identified the promoter region of LINC01119 in NCBI. Then, potential transcription factors were predicted using UCSC data, with KLF9, KLF4, SOX2, and EBF3 determined as the top 4 transcription factors (Fig. 9A). Simultaneously, we used the Lisa database to predict possible transcription factors; EBF3 was the only common transcription factor observed in the two database results (Fig. 9B). Binding sites and motif diagrams of EBF3 were predicted in the Jaspar database (Fig. 9C). Finally, we performed ChIP-PCR experiments and found that EBF3 directly regulates LINC01119 expression, knockdown of EBF3 supressed the binding of EBF3 and LINC01119 (Fig. 9D, E and Additional file 7, Additional file 8B).

## Discussion

hBMSCs have multiple differentiation capabilities and can differentiate into several tissues, such as bone, fat, and cartilage, under specific conditions [14]. Growing evidence has confirmed that the osteogenic differentiation of BMSCs, especially the inverse relationship between osteogenesis and adipogenesis of BMSCs, is essential for bone homeostasis [15, 16]. Thus, it is necessary to elucidate the molecular mechanism underlying the osteogenic differentiation of BMSCs, which could provide potential targets for improving therapeutic approaches targeting bone metabolism-related diseases, such as osteoporosis.

LncRNAs are involved in cell proliferation and development by controlling the fate of stem cells, orchestrating a complex network of interactions with regulatory proteins and other RNAs [17, 18]. Notably, several lncRNAs reportedly regulate stem cell differentiation, including LOXL1-AS1 and IMFNCR in adipocyte differentiation [19, 20], and ROCR in chondrocyte differentiation [21]. LncRNAs with potential roles in osteoblast differentiation have been previously identified, including TUG1, XIST, and GHET1 [22, 23]. However, few studies have analyzed lncRNA expression profiles of MSCs, the primary source of osteoblasts in vivo. In the present study, we analyzed the GSE80614 dataset, which consists of data regarding gene expression profiles during the early stage of human BMSC osteogenic differentiation. We compared DELs at 12 h, 1 d, 2 d, and 3 d with that observed at 0 h. LINC01119 was the only common differentially expressed lncRNA and was markedly downregulated.

In recent study, LINC01119 has been reported in adipogenic differentiation of ASCs. Chen reported that LINC01119 may have anti-adipose differentiation potential and lower expressed in adipogenic differentiation cells compared with undifferentiated human ASCs. They predicted LINC01119 may co-express with PTPRB to regulated adipocyte differentiation [24]. We identified LINC01119 as a negative regulator of the osteogenic differentiation of MSCs in our result, LINC01119 depletion markedly induced calcium deposition in BMSCs and promoted the osteogenic differentiation of MSCs. These findings present a notable therapeutic target for the clinical evaluation of bone metabolism-related diseases.

It has been reported that many signaling pathways critically impact osteogenic differentiation of BMSCs and bone metabolism [25, 26]. Herein, we used DAVID for the KEGG pathway analyses of the GSE80614 dataset and identified the involvement of the Wnt, PI3K/AKT and MAPK signaling pathways in osteogenic differentiation. Many reports have suggested that signaling pathways governing MSC osteogenic differentiation were mainly Wnt signaling, Hedgehog signaling, BMP signaling, Notch signaling, IGF signaling and MAPK signaling [15, 26]. The PI3K/AKT signaling pathway was mainly involved in the proliferation of MSC [27]. We found LINC01119 had slight effect on PI3K and MAPK signaling pathway in our study, so we selected Wnt signaling pathway for further validation. Cis and Trans analyses were performed to predict target genes of LINC01119, as shown in Fig. 6A. 10 mRNAs marked red were upregulated, whereas 13 marked blue were downregulated, when LINC01119 was downregulated. FZD4, GPC4 and BAMBI were upregulated, and SMAD3, CCND1, JUN and FOSL1 were downregulated; these mRNAs were involved in wnt signaling pathway. Because wnt signal can promote osteogenic differentiation of MSC, we choose the target gene from the upregulated mRNAs. Finally we selected FZD4 for validation, it is a receptor in the Wnt signaling pathway. RT-PCR and western blot analyses revealed that LINC01119 could suppress the transcriptional activity of the Wnt signaling pathway by targeting FZD4. LINC01119 knockdown significantly increased the mRNA expression of FZD4. RNA-EMSA also demonstrated LINC01119 can interact with FZD4, so LINC01119 might negatively regulate the osteogenic differentiation of BMSCs by targeting FZD4.

A previous study has reported that FZD4 explicitly activates the Wnt signaling pathway and promotes the osteogenic differentiation of BMSCs [28]. miR-1292 overexpression reportedly accelerates human adipose-derived stem cell (hADSC) senescence and suppresses osteogenesis. Mechanistically, FZD4 was identified as a potential target of miR-1292; miR-1292 regulates hADSC senescence and osteogenesis through the Wnt/β-catenin signaling pathway by targeting FZD4 [29]. We also observed that high FZD4 expression markedly induced calcium deposition in BMSCs. FZD4 overexpression significantly increased the mRNA expression levels of RUNX2, OSX, and OCN, as well as ALP activity. On utilizing shRNAs to silence LINC01119 and FZD4 simultaneously, we observed that low LINC01119 expression markedly induced calcium deposition in BMSCs; FZD4 knockdown alleviated this calcium deposition effect of LINC01119. Similarly, FZD4 knockdown suppressed the promoting effect induced by LINC01119 knockdown on the mRNA expression levels of RUNX2, OSX, and OCN and ALP activity. Accordingly, our findings suggest that LINC01119 inhibits osteogenic differentiation of BMSCs by targeting FZD4.

Diverse mechanisms are reportedly involved in the upstream regulation of lncRNAs, including chromosome deletion [30], transcriptional regulation by transcription factors or epigenetics [31], and post-transcriptional destabilization [32]. The loss of TSLNC8 is highly associated with the malignant features of hepatocellular carcinoma (HCC) and serves as a prognostic indicator for patients with HCC; TSLNC8 is located on the frequently deleted chromosome 8p12 [30]. In esophageal squamous cell carcinoma cell lines, LINC01503 expression increased cell proliferation, colony formation, migration, and invasion. The transcription factor TP63 binds to the super-enhancer at the LINC01503 locus, activating its transcription [31]. LncRNA OCC-1 can regulate post-transcriptional levels of numerous mRNAs by modulating RBP HuR stability [32]. In the present study, we identified the promoter region of LINC01119 in NCBI, with potential transcription factors predicted using UCSC data and the Lisa database. Next, we identified EBF3 as the transcription factor and performed ChIP-PCR experiments to demonstrate that EBF3 directly binds to the promoter and regulates LINC01119 expression. EBF3 reportedly plays an important role in osteogenesis and hematopoiesis. In chemokine ligand 12-abundant reticular cells, loss of EBF3 impairs hematopoietic stem cell numbers and leads to bone formation [33]. In mice, EBF3 knockdown did not impact chondrogenesis but led to sternum ossification defects owing to the defective generation of Runx2^+^ pre-osteoblasts [34]; these reports corroborated our results.

## Conclusion

Herein, we identified an evolutionarily conserved lncRNA expressed during MSC osteogenic differentiation, termed LINC01119. We first identified LINC01119 as a negative regulator for osteogenesis of MSCs and demonstrated the underlying molecular basis via which LINC01119 regulates osteogenesis through the Wnt pathway by targeting FZD4. Furthermore, we revealed that transcription factor EBF3 could directly bind the promoter site of LINC01119, which could provide novel insights for future exploration of osteogenic differentiation and osteoporosis.

## Supplementary Information

**Additional file 1**. The data of differentially expressed genes (DEGs) in osteogenic differentiation of MSC at 12h with that at 0 h. the result identified 592 DEGs in this group.

**Additional file 2**. The data of differentially expressed genes (DEGs) in osteogenic differentiation of MSC at 1 d with that at 0 h. the result identified 762 DEGs in this group.

**Additional file 3**. The data of differentially expressed genes (DEGs) in osteogenic differentiation of MSC at 2d with that at 0 h. the result identified 1291 DEGs in this group.

**Additional file 4**. The data of differentially expressed genes (DEGs) in osteogenic differentiation of MSC at 3d with that at 0 h. the result identified 1793 DEGs in this group.

**Additional file 5**. LINC01119 hardly regulates the BMSCs osteogenic differentiation via the PI3K/AKT or MAPK signalings. Western blot analyses and quantitative analyses of the p-JNK and p-mTOR in protein expression after transfection with sh-LINC01119 or pLV-LINC01119.

**Additional file 6**. Full-length RNA-EMSA assay of LINC01119-FZD4 interactions, the result demonstrated LINC01119 can interact with FZD4. This diagram illustrates the targeting relationship between LINC01119 and FZD4. For the purpose of logic and clarification, only the results of the control group, experimental group and cold competition group are shown.

**Additional file 7**. Full-length Chromatin immunoprecipitation (ChIP) assay for EBF3 and LINC01119, and we divided the cells into normal group (C) and EBF3 knockout group (sh) during the implementation of the experiment. Data from the normal group showed that EBF3 could bind to LINC01119, but this binding decreased when EBF3 was knocked out, which indirectly proved that EBF3 could directly bind to LINC01119.

**Additional file 8**. (A) The explanation of figure 6C and additional file 6. (B) The explanation of figure 9E and additional file 7.

## Data Availability

The datasets for this study are available from the corresponding author on reasonable request.

## Abbreviations

MSCs: Mesenchymal stem cells
lncRNAs: Long non-coding ribonucleic acids
BMSCs: Bone marrow mesenchymal stem cells
hBMSC: Human bone marrow mesenchymal stem cells
DMEM: Dulbecco's modified Eagle’s medium
FBS: Fetal bovine serum
GEO: Gene Expression Omnibus
DEGs: Differentially expressed genes
FC: Fold change
GSEA: Gene set enrichment analysis
GO: Gene Ontology
KEGG: Kyoto Encyclopedia of Genes and Genomes
qRT-PCR: Quantitative real-time PCR
ALP: Alkaline phosphatase
ARS: Alizarin red S
PBS: Phosphate-buffered saline
ChIP: Chromatin immunoprecipitation
FISH: Fluorescence in situ hybridization
SD: standard deviation
ANOVA: Analysis of variance
hADSC: Human adipose-derived stem cell
HCC: Hepatocellular carcinoma
