# Supplementary figures and images for: LINC01119 negatively regulates osteogenic differentiation of mesenchymal stem cells via the Wnt pathway by targeting FZD4
Source: Stem Cell Res Ther. 2022 Jan 29;13:43. doi: 10.1186/s13287-022-02726-1 (PMC8800246; doi:10.1186/s13287-022-02726-1)

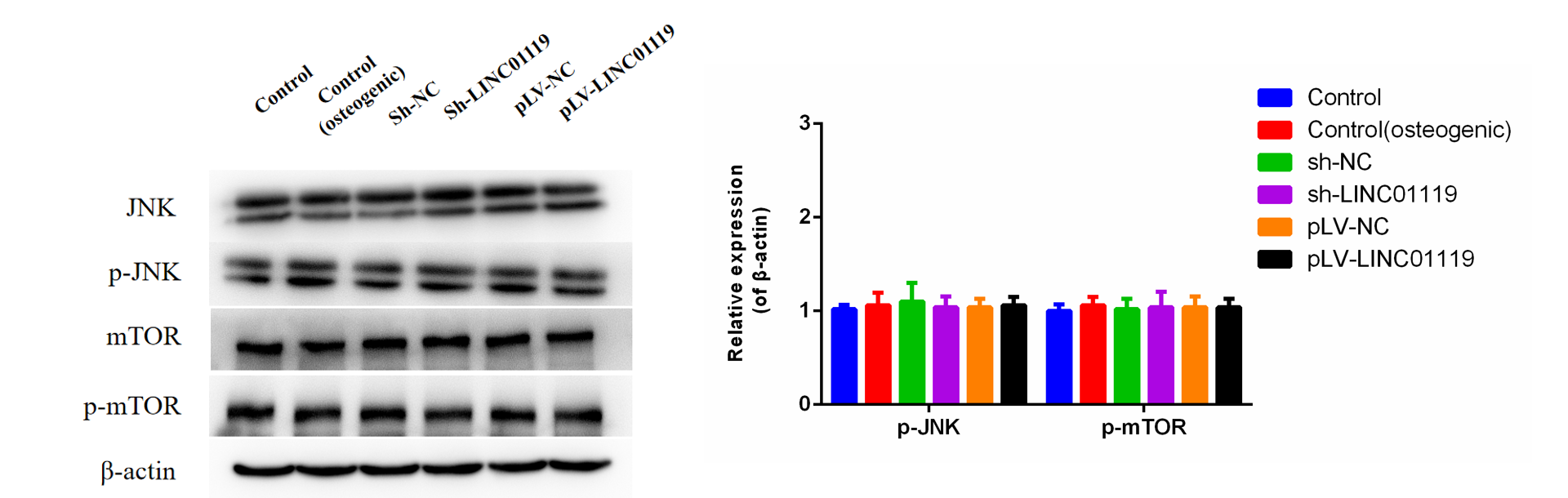

Supplement: Supplementary file 5 — Additional file 5. LINC01119 hardly regulates the BMSCs osteogenic differentiation via the PI3K/AKT or MAPK signalings. Western blot analyses and quantitative analyses of the p-JNK and p-mTOR in protein expression after transfection with sh-LINC01119 or pLV-LINC01119. [file 13287_2022_2726_MOESM5_ESM.tif]

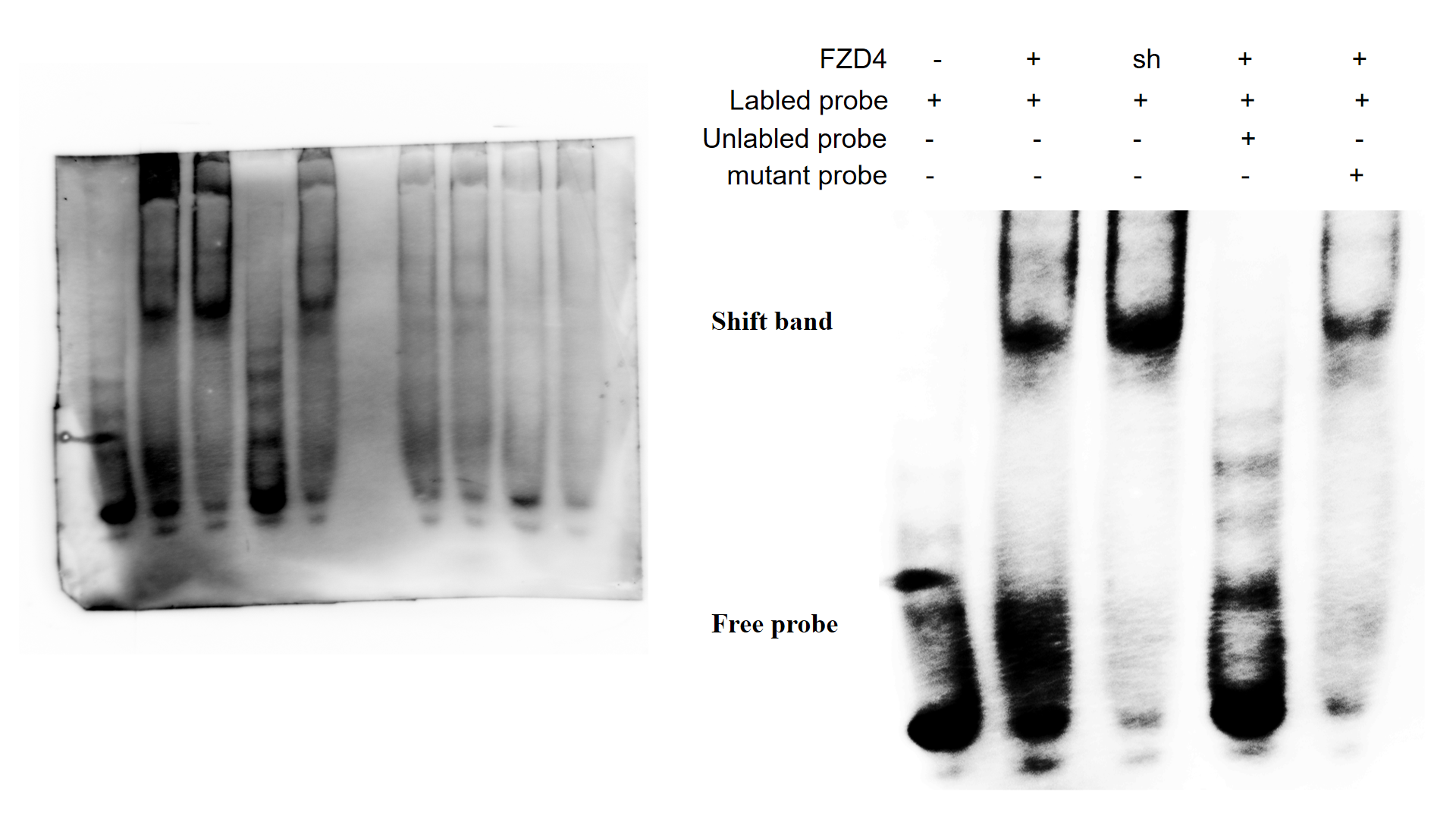

Supplement: Supplementary file 6 — Additional file 6. Full-length RNA-EMSA assay of LINC01119-FZD4 interactions, the result demonstrated LINC01119 can interact with FZD4. This diagram illustrates the targeting relationship between LINC01119 and FZD4. For the purpose of logic and clarification, only the results of the control group, experimental group and cold competition group are shown. [file 13287_2022_2726_MOESM6_ESM.tif]

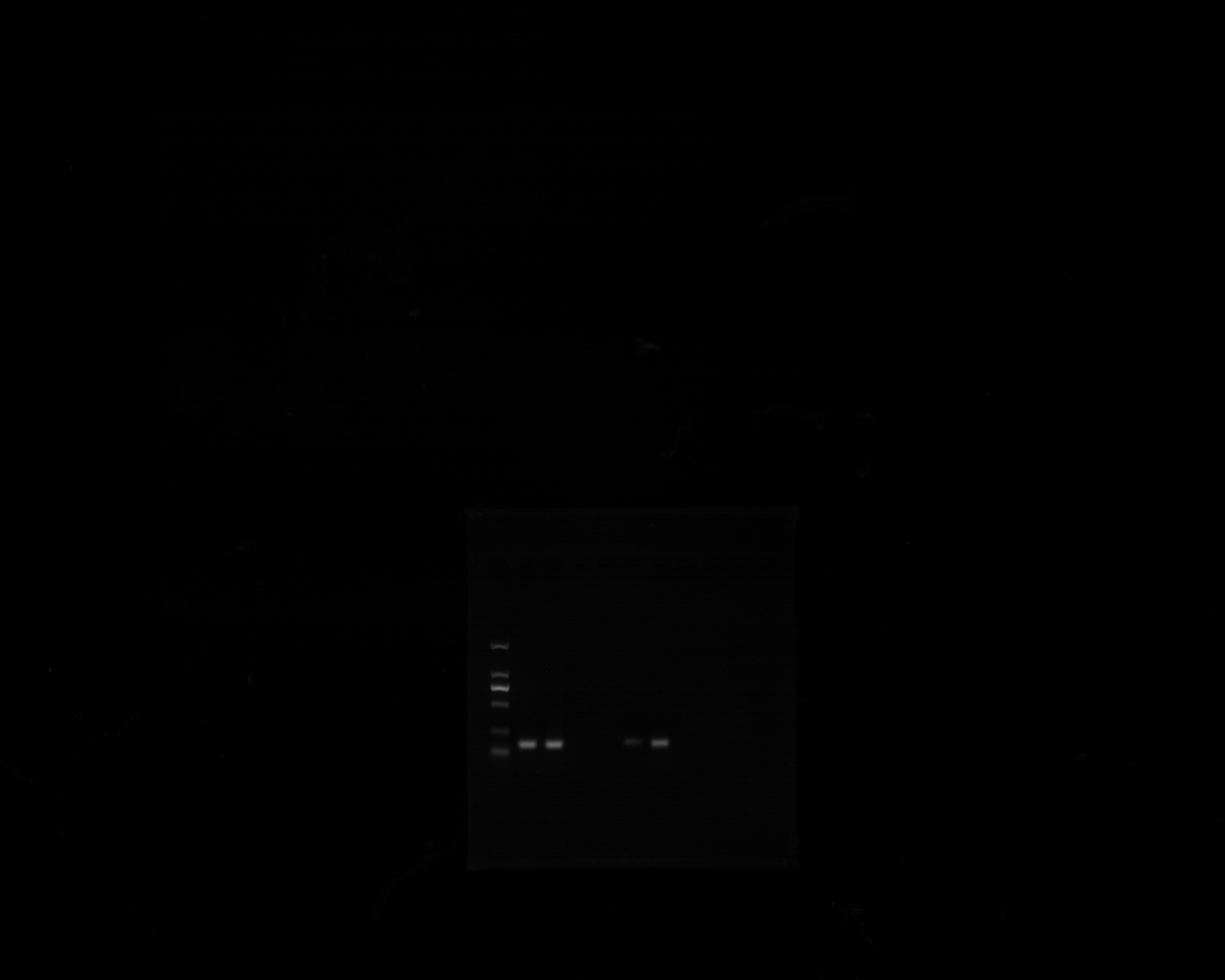

Supplement: Supplementary file 7 — Additional file 7. Full-length Chromatin immunoprecipitation (ChIP) assay for EBF3 and LINC01119, and we divided the cells into normal group (C) and EBF3 knockout group (sh) during the implementation of the experiment. Data from the normal group showed that EBF3 could bind to LINC01119, but this binding decreased when EBF3 was knocked out, which indirectly proved that EBF3 could directly bind to LINC01119. [file 13287_2022_2726_MOESM7_ESM.tif]
