# Supplementary material for: LINC01119 negatively regulates osteogenic differentiation of mesenchymal stem cells via the Wnt pathway by targeting FZD4
Source: Stem Cell Res Ther. 2022 Jan 29;13:43. doi: 10.1186/s13287-022-02726-1 (PMC8800246; doi:10.1186/s13287-022-02726-1)

**Explanation of two gels**

Dear editors,

We submitted the complete gels about EMSA and CHIP, and we wanted to make some explanation about the two gels as follows.

(A) According to the comments of reviewers, CLIP is suggested to be added, but duo to technical and time constraints, we performed EMSA to explain the interaction between lncRNA and protein. In the experiment, we designed control group, experimental group, competition group (containing specific competitor) and mutation group (containing competitor). In addition, another group of FZD4 protein was added, which was extracted from the sh-LINC01119 cells, namely the third result in the figure below. The results showed that this group of FZD4 protein was more obvious, which also indirectly indicated that the expression of FZD4 increased after LINC01119 knockdown, resulting in the increase of FZD4 protein quantity and more obvious bands. However, when writing in the text, we found that the result of grouping disrupted the logic of the article, because the variation relationship between LINC01119 and FZD4 was mentioned later in the article, the targeting relationship between LINC01119 and FZD4 was only explained here. Therefore, we thought the third result in the figure below was not suitable here, and from the point of view of the logicality of the article and being able to clarify the problem, we only added the results of the control group, the experimental group and competition group in the article. we added the complete gel as the additional file.We feel apologized for the trouble caused! Thank you!


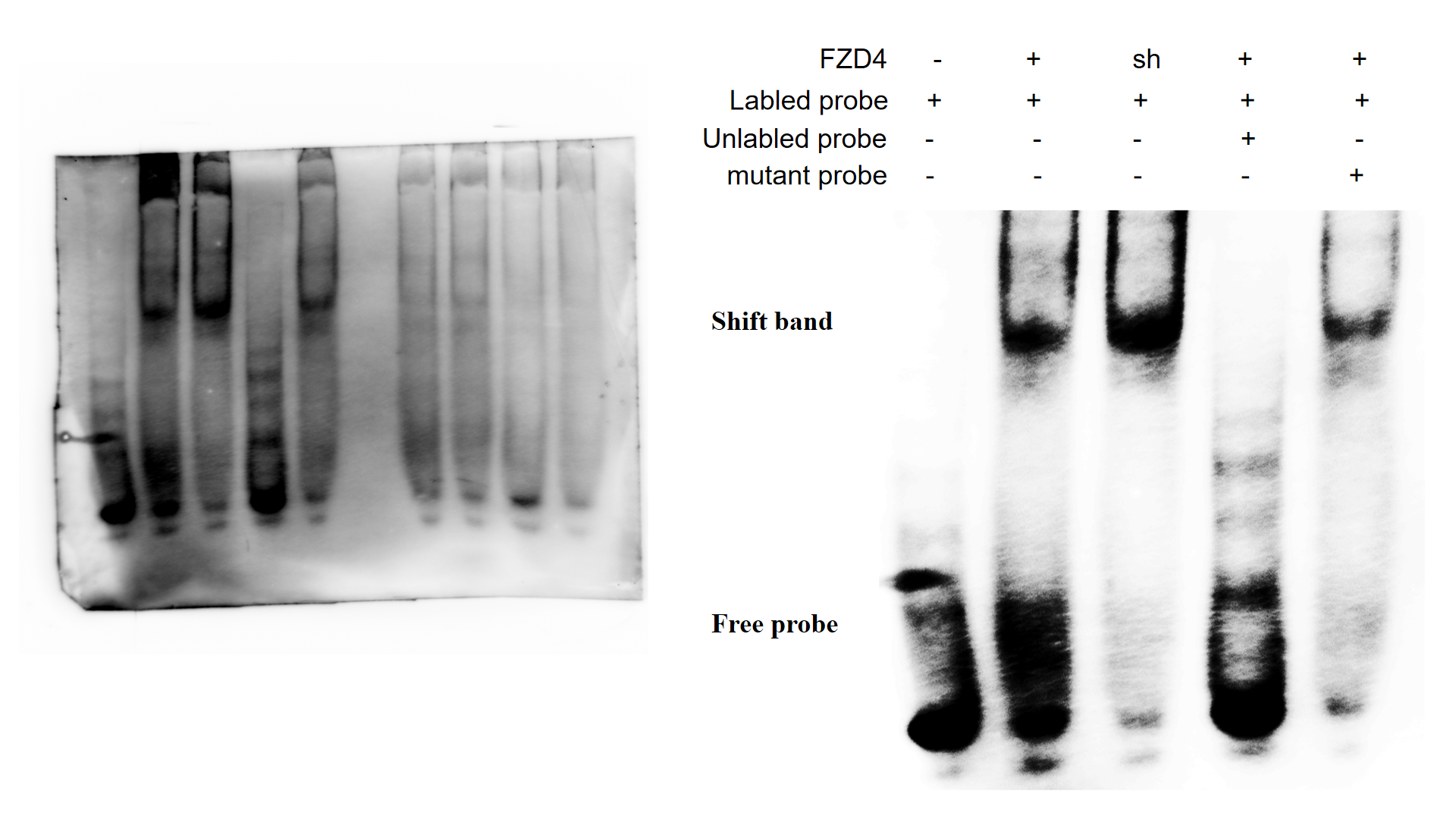


(B) In response to the question of CHIP standardization raised by reviewers, we made the latest CHIP results and divided the cells into normal group (C) and EBF3 knockdown group (sh) during the implementation of the experiment. Data from the normal group showed that EBF3 could bind to LINC01119, but this binding decreased when EBF3 was knocked down, which indirectly proved that EBF3 could directly bind to LINC01119. However, we thought the results should be more concise, clear and easy to understand, so the results we submitted last time only showed the results of the normal group, which showed that EBF3 could be combined with LINC01119. Finally, after discussion by our research group, we thought it would be more complete and reasonable to present the complete results to everyone, so we resubmitted the complete results and the complete gel was added as the additional file. We apologize for the trouble caused by our work!


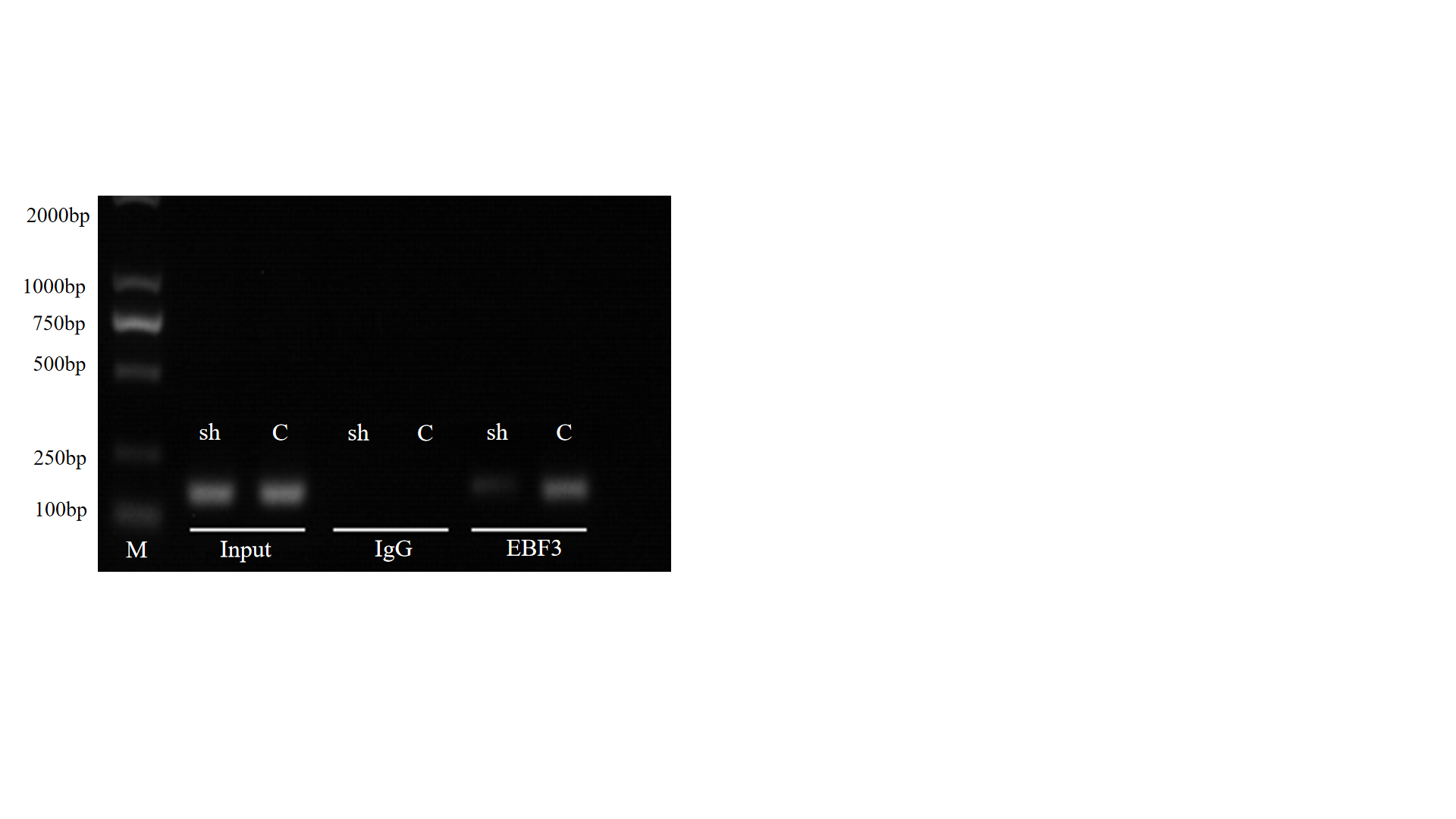

Supplement: Supplementary file 8 — Additional file 8. (A) The explanation of figure 6C and additional file 6. (B) The explanation of figure 9E and additional file 7. [file 13287_2022_2726_MOESM8_ESM.doc]
